# The genetic polymorphisms of *ZC3HC1* and *SMARCA4* are associated with hypertension risk

**DOI:** 10.1002/mgg3.942

**Published:** 2019-09-10

**Authors:** Huijun Ma, Yongjun He, Mei Bai, Linhao Zhu, Xue He, Li Wang, Tianbo Jin

**Affiliations:** ^1^ Department of Cardiology The First Hospital of Xi'an Xi'an China; ^2^ Key Laboratory of Molecular Mechanism and Intervention Research for Plateau Diseases of Tibet Autonomous Region Xianyang Shaanxi China; ^3^ Key Laboratory of High Altitude Environment and Genes Related to Diseases of Tibet Autonomous Region Xianyang Shaanxi China; ^4^ Key Laboratory for Basic Life Science Research of Tibet Autonomous Region, School of Medicine Xizang Minzu University Xianyang Shaanxi China; ^5^ Key Laboratory of Resource Biology and Biotechnology in Western China (Northwest University), Ministry of Education, School of Life Sciences Northwest University Xi'an Shaanxi China

**Keywords:** case–control study, genetics polymorphisms, hypertension, *SMARCA4*, *ZC3HC1*

## Abstract

**Aim:**

In this study, we aimed to evaluate the association between genetic variants of *ZC3HC1* and *SMARCA4* and hypertension risk in the Chinese Han population.

**Methods:**

The Agena MassAssary platform was used to determine the genotypes of eight SNPs in *ZC3HC1* and *SMARCA4* from 350 hypertension patients and 483 healthy controls. Chi‐squared tests and genetic model were used to evaluate the associations. Odds ratios and 95% confidence intervals were calculated using unconditional logistic regression. The statistical power of this study was estimated through the Power and Sample Size Calculation online software.

**Result:**

In the genetic model analysis, we identified that the SNP of rs1464890 in *ZC3HC1* was associated with a 0.68‐fold decreased risk of hypertension in the codominant model and 0.65‐fold decreased risk in the dominant model. Rs4507692 in *ZC3HC1* was associated with a 0.69‐fold decreased risk of hypertension in the codominant model and 0.66‐fold decreased risk in the dominant model. The genotype “G/A‐A/A” of rs11879293 and the genotype “G/T‐T/T” of rs1122608 in *SMARCA4* were significantly associated with decreasing the hypertension risk. In addition, the “A_rs2242487_ T_rs1464890_ T_rs4507692_” *ZC3HC1* haplotype was associated with a decreased risk of hypertension.

**Conclusion:**

The present study suggested that *ZC3HC1* and *SMARCA4* polymorphism may conducive to play a protective role against the hypertension risk.

## 1 INTRODUCTION

Hypertension is the main factor for morbidity and mortality worldwide (Kearney et al., 2005; Staessen, Wang, Bianchi, & Birkenhager, 2003; Stokes, Kannel, Wolf, D'Agostino, & Cupples, 1989). However, the specific pathogenesis of hypertension is still unclear. (Mein, Caulfield, Dobson, & Munroe, 2004). Several studies have shown that the etiology and pathogenesis of hypertension are likely to comprise a multifactorial disorder resulting from environmental factors (overweight, alcohol, and smoke) and genetic factors or their interaction (Carretero & Oparil, 2000; Lu et al., 2015). Recently, hypertension has been found to be the main factor in the occurrence of myocardial infarction, stroke, cardiac and renal failure and later lesions of the retina of the eyes (Mosterd et al., 1999), and that has also been steadily increasing in China for the past several years.

Hypertension can reduce the function of which is easy to form atherosclerosis (Fujimaki et al., 2015), and atherosclerosis is the main pathological basis of coronary heart disease (Lefevre & Puymirat, 2017; Savoia et al., 2017). *SMARCA4* (OMIM: 603254) and *ZC3HC1* (OMIM: 603254) are high‐risk genes for coronary heart disease. At present, there are many researches about the association between *SMARCA4* and *ZC3HC1* and coronary heart disease. For examples, GWAS study showed that *SMARCA4* was related to coronary heart disease (Kathiresan, Voight, et al., 2009) and myocardial infarction (Martinelli et al., 2010). Previous studies were also identified rs11879293, rs12232780, rs2072382, and rs1529729 variants' effect on hypertension and dyslipidemia‐related disease (Fujimaki et al., 2015; Liu et al., 2011). Guo et al. (2017) found the variant in the *SMARCA4* was associated with coronary heart disease susceptibility in Han Chinese population. Linseman et al. (2017) identified that *ZC3HC1* was associated with protection from coronary artery disease. However, few studies have examined the association between *SMARCA4* and *ZC3HC1* and hypertension risk. For the current study, we evaluated the association between eight SNPs in *SMARCA4* and *ZC3HC1* and hypertension risk, and aimed to find the relations of these SNPs and hypertension risk in Han Chinese population.

## 2 MATERIALS AND METHODS

### 2.1 Ethics statement

This investigation was conducted in accordance with the ethical standards of the Declaration of Helsinki and following the national and international guidelines. Additionally, the protocol of this study was approved by the ethics committee of the first affiliated Hospital of Xi'an Jiaotong University. Written informed consent was obtained from all the participants after a full explanation of the study. The experimental protocol was implemented in accordance with the approved guidelines.

### 2.2 Subjects

We recruited a total of 350 patients, which were diagnosed with hypertension, and were enrolled from Northwestern China at the first affiliated Hospital of Xi'an Jiaotong University. The controls were 483 healthy subjects recruited from routine healthy examinations in the same hospital. All subjects were from the Chinese Han population living in Shaanxi province. Hypertensive subjects were defined as having a systolic blood pressure (SBP) of at least 140 mmHg and a diastolic blood pressure (DBP) of at least 90 mmHg (de Menezes, Oliveira, & Ma, 2014). All the hypertensive patients were not only required to be free of other cardiovascular diseases, metabolic diseases, cancers or familial hereditary disease, but diagnosed with hypertension before the age of 70 years. Normotensive controls were recruited from the same hospital. These individuals were never treated with antihypertensive medications, and their SBP were less than 140 mmHg and DBP less than 90 mmHg. They had no family history of hypertension.

### 2.3 SNP selection and genotyping

Eight SNPs in *ZC3HC1* and *SMARCA4* had minor allele frequencies greater than 5% in the 1000 Genomes Project (http://www.internationalgenome.org/). A GoldMag‐Mini Purification Kit (GoldMag Co. Ltd.) was performed to extract genomic DNA from whole blood. DNAs were stored at −80°C until analysis. DNA concentrations were measured using a NanoDrop 2000 (Thermo Scientific). The primers were designed online (https://agenacx.com/online-tools/). Agena MassARRAY Assay Design 4.0 software was used to design multiplexed SNP MassEXTEND assay, and SNP genotyping was performed utilizing the Agena MassARRAY RS1000 as recommended by the manufacturer. Agena Typer 4.0 software was used to perform data management and analyses.

### 2.4 Statistical analysis

All statistical analyses were performed using Microsoft Excel and SPSS 19.0 (SPSS). All *p* values were two‐sided (*p* *< *.05 was considered as achieving the threshold of statistical significance). Each SNP frequency in the control subjects was tested by deviation from Hardy–Weinberg equilibrium by the Fisher's test. Allele frequencies and genotype frequencies for each SNP in cases and controls were compared by the chi‐squared test/Fisher's exact test to determine the associations between genotypes and hypertension risk. Odds ratio (OR) values and 95% confidence intervals (CIs) measured the risk allele effect size using unconditional logistic regression analysis (Bland & Altman, 2000). Four genetic models (codominant, dominant, recessive, and log‐additive) were used to evaluate the potential association of *ZC3HC1* and *SMARCA4* polymorphisms with risk and clinical parameters of hypertension. Finally, the Haploview was used to construct haplotype and genetic association at significant polymorphism loci and to estimate the pairwise linkage disequilibrium (LD), haplotype software (version4.2) and SHEsis software platform (http://www.nhgg.org/analysis/) ction, and genetic association at polymorphism loci (Barrett, Fry, Maller, & Daly, 2005; Shi & He, 2005). Power and Sample Size Calculation software (http://biostat.mc.vanderbilt.edu/wiki/Main/PowerSampleSize) was used to calculate the power of the significant difference.

## 3 RESULTS

### 3.1 Characteristics of the participants

This study involved 889 subjects, including 350 patients (204 males and 146 females; age at diagnosis: 62.68 ± 10.7 years) and 483 healthy controls (183 males and 300 females; age: 50.37 ± 7.9 years).There were statistical differences in age and sex distribution between the case and control groups (Table 1).

**Table 1:** General characteristics of the study population

variable	Cases (*n* = 350)	%	Controls (*n* = 483)	%	*p* value
Gender					<.001^a^
Male	204	58.3	183	37.9	
Female	146	41.7	300	62.1	
Age, yr (mean ± *SD*)	62.68 ± 10.7		50.37 ± 7.9		<.01^b^

^a^ *p* values were calculated by Student's *t* tests.

^b^ *p* values were calculated from two‐sided chi‐squared tests.

### 3.2 The associations between ZC3HC1 and SMARCA4 SNPs and hypertension

Eight SNPs in *ZC3HC1* and *SMARCA4* were selected. Position, alleles, and minor allele frequency of these SNPs were showed in Table 2. One SNP (rs2072382) was excluded for significant deviation from Hardy–Weinberg equilibrium (*p* < .05), a chi‐square analysis revealed that no significant differences in allele frequency distributions of SNPs between the hypertension patients group and the healthy control analyzed. In other words, there is no statistically significant association between allele and hypertension risk.

**Table 2:** Allele frequencies in cases and controls and odds ratio estimates for hypertension risk

SNP	Gene(s)	Locus	Alleles (A/B)	MAF		*p* values	OR (95%CI)	*p* a value	*p* b value
				Case	Control				
rs2242487	*ZC3HC1*	7q32.2	A/G	0.233	0.270	0.249	0.82 (0.66–1.03)	.088	.011
rs1464890	*ZC3HC1*	7q32.2	T/C	0.277	0.314	0.247	0.94 (0.68–1.04)	.102	.013
rs4507692	*ZC3HC1*	7q32.2	T/C	0.277	0.314	0.246	0.84 (0.68–1.04)	.108	.014
rs11879293	*SMARCA4*	19p13.2	A/G	0.237	0.259	0.097	0.89 (0.71–1.12)	.313	.039
rs12232780	*SMARCA4*	19p13.2	A/G	0.199	0.213	0.135	0.91 (0.72–1.16)	.466	.058
rs2072382	*SMARCA4*	19p13.2	T/C	0.337	0.280	**0.018** #	1.31 (1.06–1.62)	.012	.002
rs1529729	*SMARCA4*	19p13.2	C/T	0.224	0.228	0.091	0.98 (0.78–1.24)	.871	.109
rs1122608	*SMARCA4*	19p13.2	T/G	0.066	0.084	0.561	0.77 (0.53–1.12)	.169	.021

Abbreviations: Alleles A/B, Minor/major alleles; CI, confidence interval; HWE, Hardy–Weinberg equilibrium; MAF, minor allele frequency; OR, odds ratio; SNP, single‐nucleotide polymorphism.

^#^Site with HWE *p* ≤ .05 excluded; ^a^ *p* values were calculated using two‐sided chi‐squared test. ^b^ *p* values were adjusted by Bonferroni correction. ^a^ *p* < .05 indicates statistical significance; ^b^ *p* < .05 indicates statistical significance.

### 3.3 Associations between genotype frequencies and hypertension risk

As shown in Table 3, logistic regression analyses revealed that the genotype “T/C” of rs1464890 in *ZC3HC1* was associated with a decreased risk of hypertension in the codominant model (OR = 0.48, 95% CI, 0.47–0.98, *p* = .044) and dominant model (OR = 0.65, 95% CI, 0.46–0.93, *p* = .016), respectively. Rs4507692 in *ZC3HC1* was associated with a 0.69‐fold and a 0.66‐fold decreased risk of hypertension under the codominant model and dominant model, respectively. The genotype “G/A‐A/A” of rs11879293 in *SMARCA4* was significantly associated with decreasing the risk of hypertension under the dominant model (OR = 0.70; 95% CI = 0.49–0.99, *p* = .044). Rs1122608 in *SMARCA4* was also significantly associated with a decreased risk of hypertension in the dominant model (OR = 0.61; 95% CI = 0.38–0.99, *p* = .047 for the “G/T‐T/T” genotype) and log‐additive model (OR = 0.61; 95% CI = 0.38–0.98, *p* = .038), respectively. Furthermore, the statistical power of our study was more than 80%.

**Table 3:** Relationships between *ZC3HC1* and *SMARCA4* polymorphism and hypertension risk

SNP	Model	Genotype	Genotype frequency		*p* a‐value	OR (95% CI)	Study power
			Control (%)	Case (%)			
*ZC3HC1*							
rs2242487	Codominant	G/G	262 (54.4	209 (59.7)	.158	1	
		A/G	180 (37.3)	119 (34.0)		0.71 (0.49–1.03)	
		A/A	40 (8.3)	22 (6.3)		0.69 (0.35–1.38)	
	Dominant	G/G	262 (54.4)	209 (59.7)	.054	1	
		A/G‐A/A	220 (45.6)	141 (40.3)		0.71 (0.50–1.01)	
	Recessive	G/G‐A/G	442 (91.7)	328 (93.7)	.479	1	
		A/A	40 (8.3)	22 (6.3)		0.78 (0.40–1.54)	
	Log‐additive	—	—	—	.069	0.77 (0.59–1.02)	
rs1464890	Codominant	C/C	232 (48.1)	183 (52.3)	**.044**	1	**.925**
		T/C	197 (40.9)	140 (40.0)		**0.68 (0.47–0.98)**	
		T/T	53 (11.0)	27 (7.7)		0.55 (0.29–1.02)	
	Dominant	C/C	232 (48.1)	183 (52.3)	**.016**	1	**.978**
		T/C‐T/T	250 (51.9)	167 (47.7)		**0.65 (0.46–0.93)**	
	Recessive	C/C‐T/C	429 (89.0)	323 (92.3)	.149	1	
		T/T	53 (11.0)	27 (7.7)		0.65 (0.35–1.18)	
	Log‐additive	—	—	—	**.014**	0.72 (0.55–0.94)	
rs4507692	Codominant	C/C	233 (48.2)	183 (52.3)	**.049**	**1**	**.905**
		C/T	197 (40.8)	140 (40.0)		**0.69 (0.48–0.99)**	
		T/T	53 (11.0)	27 (7.7)		0.55 (0.29–1.03)	
	Dominant	C/C	233 (48.2)	183 (52.3)	**.019**	**1**	**.969**
		C/T‐T/T	250 (51.8)	167 (47.7)		**0.66 (0.47–0.93)**	
	Recessive	C/C‐C/T	430 (89)	323 (92.3)	0.158	1	
		T/T	53 (11.0)	27 (7.7)		0.65 (0.36–1.19)	
	Log‐additive	—	—	—	**.015**	**0.72 (0.55–0.94)**	
*SMARCA4*							
rs11879293	Codominant	G/G	258 (53.4)	204 (58.3)	.110	1	
		G/A	200 (41.4)	126 (36.0)		0.68 (0.47–0.97)	
		A/A	25 (5.2)	20 (5.7)		0.87 (0.41–1.81)	
	Dominant	G/G	258 (53.4)	204 (58.3)	**.044**	1	**.898**
		G/A‐A/A	225 (46.6)	146 (41.7)		**0.70 (0.49–0.99)**	
	Recessive	G/G‐G/A	458 (94.8)	330 (94.3)	.960	1	
		A/A	25 (5.2)	20 (5.7)		1.02 (0.49–2.09)	
	Log‐additive	—	—	—	.101	0.79 (0.59–1.05)	
rs12232780	Codominant	G/G	293 (60.7)	221 (63.1)	.210	1	
		G/A	174 (36.0)	119 (34.0)		0.72 (0.50–1.04)	
		A/A	16 (3.3)	10 (2.9)		0.72 (0.26–2.00)	
	Dominant	G/G	293 (60.7)	221 (63.1)	.073	1	
		G/A‐A/A	190 (39.3)	129 (36.9)		0.72 (0.51–1.03)	
	Recessive	G/G‐G/A	467 (96.7)	340 (97.1)	.678	1	
		A/A	16 (3.3)	10 (2.9)		0.81 (0.30–2.22)	
	Log‐additive	—	—	—	.080	0.76 (0.55–1.04)	
rs1529729	Codominant	T/T	280 (58.2)	205 (58.6)	.772	1	
		T/C	183 (38.0)	133 (38.0)		0.98 (0.69–1.39)	
		C/C	18 (3.7)	12 (3.4)		0.71 (0.28–1.80)	
	Dominant	T/T	280 (58.2)	205 (58.6)	.781	1	
		T/C‐C/C	201 (41.8)	145 (41.4)		0.95 (0.67–1.34)	
	Recessive	T/T‐T/C	463 (96.3)	338 (96.6)	.469	1	
		C/C	18 (3.7)	12 (3.4)		0.72 (0.28–1.80)	
	Log‐additive	—	—	—	.630	0.93 (0.69–1.25)	
rs1122608	Codominant	G/G	404 (83.6)	304 (86.9)	.081	1	
		G/T	77 (15.9)	46 (13.1)		0.63 (0.38–1.02)	
		T/T	2 (0.4)	0 (0.0)		0.00 (0.00‐NA)	
	Dominant	G/G	404 (83.6)	304 (86.9)	**.047**	1	**.846**
		G/T‐T/T	79 (16.4)	46 (13.1)		**0.61 (0.38–0.99)**	
	Recessive	G/G‐G/T	481 (99.6)	350 (100.0)	.252	1	
		T/T	2 (0.4)	0 (0.0)		0.00 (0.00‐NA)	
	Log‐additive	—	—	—	**.038**	**0.61 (0.38–0.98)**	

Note

The bold values and *p < *.05 indicate statistical significance.

Abbreviations: OR, odds ratio; SNP, single nucleotide polymorphism; 95% CI, 95% confidence interval.

a *p* values were calculated by unconditional logistic regression analysis with adjustments for age and gender.

### 3.4 Associations between haplotype analyses and hypertension risk

**Figure 1:**
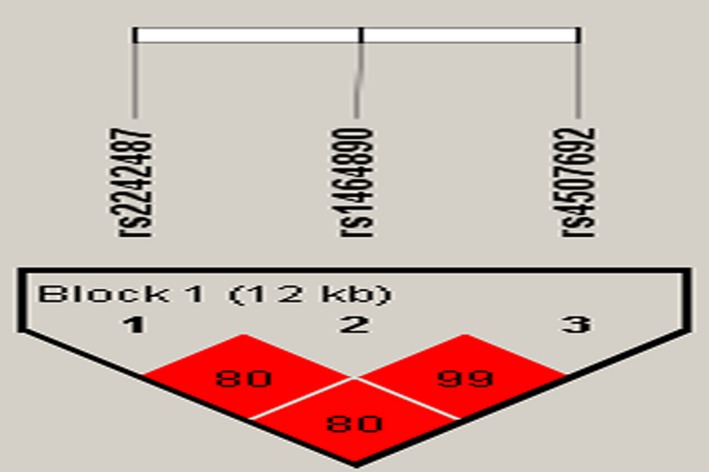
Linkage disequilibrium (LD) plots containing four SNPs from *ZC3HC1*

Linkage disequilibrium and haplotype analyses of the SNPs in the case and control samples were further studied. Haplotype analysis detected the block in *ZC3HC1* (Figure 1). Rs2242487, rs1464890, and rs4507692 had very strong linkage disequilibria; compared to the “GCC” wild‐type, the haplotype ‘‘ATT’’ was associated with a decreased risk of hypertension (OR = 0.75; 95% CI = 0.56–0.99; *p* = .044) after adjustments for age and gender (Table 4).

**Table 4:** Haplotype analysis results in this study

Haplotypes				Without adjusted		With adjusted	
rs2242487	rs1464890	rs4507692	Freq	OR (95% CI)	*p*‐value	OR (95% CI)	*p* a‐value
G	C	C	0.701	1	—	1	—
A	T	T	0.253	0.83 (0.66–1.03)	.095	**0.75 (0.56–0.99)**	**.044**
G	T	T	0.045	0.94 (0.58–1.53)	.810	0.55 (0.29–1.02)	.056

Note

The bold values and *p < *.05 indicate statistical significance.

Abbreviations: CI, confidence interval; OR, odds ratio; SNP, single nucleotide polymorphism.

a *p*: Adjusted by gender and age.

## 4 DISCUSSION

Genetic studies have provided insight into numerous diseases, including hypertension. Eight SNPs in *ZC3HC1* and *SMARCA4* have been investigated in other diseases. In this study, we examined 833 subjects (350 patients with hypertension and 483 healthy controls) to determine whether they were associated with the risk of hypertension in the Han Chinese population. Our results suggest that rs1464890 and rs4507692 (*ZC3HC1*), rs11879293 (*SMARCA4*) and rs1122608 (*SMARCA4*) were conducive to play a protective role to against the risk of hypertension. In addition, the “ATT” *ZC3HC1* haplotype was associated with a 0.75‐fold decreased risk of hypertension.

*ZC3HC1* (zinc finger, C3HC‐type containing 1) was also called NIPA (nuclear interaction partner of ALK), which could monitor the timing of mitotic entry and was thought to contribute to the development of carcinogenesis together with oncogenic proteins (Li & Morris, 2008). Studies have been shown that mediators of angiogenesis may play an important role in the regulation of endothelial integrity and inflammation and it was possible that changes in the stability and functional properties of *ZC3HC1* protein may play a role in the endothelial dysfunction (Schunkert et al., 2011), especially in the coronary heart disease and hypertension. Recently, a genome‐wide association study, reported by Linseman et al. (2017), found that *ZC3HC1* polymorphism was associated with a protective role in coronary artery disease. Kunnas and Nikkari (2015) reported the association of *ZC3HC1* rs11556924 genetic variant with hypertension in a Finnish population. However, in previous studies, many reports only focused on the association of genetic variant in *ZC3HC1* (rs11556924) with diseases, the genetic polymorphism of other locus in *ZC3HC1* were little reported. Therefore, in our research, we studied the relationship between *ZC3HC1* SNPs (rs2242487, rs1464890, and rs4507892) and hypertension in Chinese Han population, and we found that the polymorphism of *ZC3HC1* (rs1464890) has a strong protective effects on the hypertension.

*SMARCA4* (also known as *BRG1*) is located in chromosomal region of 19p13.2, and its protein is the important catalytic component of the SWI/SNF complexes (Moes‐Sosnowska et al., 2015). It is composed of multiple domains, a conserved C‐terminal bromodomain, the less characterized N‐terminal region which has crucial effect on DNA binding, recruitment of SWI/SNF, and the recognition of modified histone proteins (Singh, D'Silva, & Holak, 2006). *SMRACA4* is located closely to the low‐density lipoprotein receptor gene and disrupting chromatin structure regulates the transcription of various genes using the chemical energy of adenosine triphosphate hydrolysis (Mulholland, Xu, Sugiyama, & Zhao, 2012). In our research, we found rs11879293 and rs1122608 in *SMARCA4* seemed to have strong protective effects on the hypertension. However, the previous studies, Guo et al. (2017) found rs11879293 was associated with decreasing the risk of coronary heart disease, and another study found rs11879293 was associated with increasing the risk of hepatocellular carcinoma is more pronounced in males, younger individuals, and nondrinkers (Pan et al., 2007). Kathiresan, Willer, et al. (2009) found the loci rs1122608 was associated with elevating the risk of low‐density lipoprotein cholesterol and coronary heart disease in Caucasian population. At present, there were no relevant reports on the relationship between rs11879293 and rs1122608 with hypertension. Therefore, in future studies, we will consider that the *SMARCA4* may function differently in varying disease mechanisms.

To sum up, in our study, we confirmed two genes (*ZC3HC1* and *SMARCA4*) are associated with risk of hypertension in Han Chinese population for the first time, which may provide new data to facilitate earlier diagnosis and promote early prevention, and shed light on the new candidate genes and new ideas for the study of subsequent occurrence mechanism of hypertension. Some potential limitations of our current study should be considered when interpreting the results. Investigating these SNPs should use more clinical data with bigger samples. Our current research is fundamental; further functional studies and larger population‐based prospective studies are required to understand the genetic factors underlying hypertension.

## CONFLICT OF INTEREST

The authors have no conflict of interest to disclose.
